# *Talaromyces marneffei* Infections in 8 Chinese Children with Inborn Errors of Immunity

**DOI:** 10.1007/s11046-022-00659-0

**Published:** 2022-09-30

**Authors:** Linlin Wang, Ying Luo, Xiaolin Li, Yixian Li, Yu Xia, Tingyan He, Yanyan Huang, Yongbin Xu, Zhi Yang, Jiayun Ling, Ruohang Weng, Xiaona Zhu, Zhongxiang Qi, Jun Yang

**Affiliations:** 1 Department of Rheumatology and Immunology, Shenzhen Children’s Hospital, 7019 Yitian Road, Shenzhen, 518026 China; 2 Shenzhen Institute of Pediatrics, Shenzhen Children’s Hospital, 7019 Yitian Road, Shenzhen, 518026 China; 3 Department of Pediatric Rheumatology and Immunology, Zhongshan Boai Hospital Affiliated to Southern Medical University, Zhongshan, 528403 China

**Keywords:** *Talaromycesmarneffei*, Inborn errors of immunity, Primary immunodeficiency diseases, Gene mutations, Children

## Abstract

**Purpose:**

*Talaromyces marneffei* (TM) is an opportunistic fungus leading to multi-organ damages and poor prognosis in immunocompromised individuals. TM infections in children are rare and our knowledge to TM infection is insufficient. To investigate the clinical characteristics of TM-infected children and to explore the underlying mechanisms for host against TM, we analysed TM-infected patients diagnosed in our hospital.

**Methods:**

Eight patients with TM infections have been identified in Shenzhen Children’s Hospital during 2017–2021. Clinical data were collected from medical records. Immunological features were evaluated by flow cytometry. Literatures were also reviewed to summarize the reported inborn errors of immunity (IEIs) with TM infections.

**Results:**

All 8 children were HIV-negative. The most common symptom of TM infections was fever (8/8), followed by weight loss (7/8), pneumonia (7/8), hepatomegaly (7/8), splenomegaly (6/8), anemia (6/8), lymphadenopathy (5/8), thrombocytopenia (3/8), diarrhea (3/8), rashes or skin lesions (3/8), and osteolytic lesions (1/8). Five children died during the follow-ups. CD3+ T cells were decreased in 6 patients. Eight patients had reduced natural killer cells. All patients went gene sequencing and were finally diagnosed as IEIs, including STAT1 gain-of-function, IL-2 receptor common gamma chain deficiency, adenosine deaminase deficiency, CD40 ligand deficiency, and STAT3 deficiency. Another 4 types of IEIs (CARD9, IFN-*γ* receptor 1, RelB, and NFKB2 deficiency), have been reported with TM infections based on literature review.

**Conclusion:**

TM infections resulted in systemic injuries and high mortality. The spectrum of IEIs underlying TM infections indicated that T cell-mediated immunity, IFN-*γ*, IL-17 signalings and NF-κB pathways were important for host responses against TM infection. In reverse, for HIV-negative children without other secondary immunodeficiencies, IEIs should be considered in TM-infected children.

**Supplementary Information:**

The online version contains supplementary material available at 10.1007/s11046-022-00659-0.

## Introduction

Inborn errors of immunity (IEIs), also known as primary immunodeficiency diseases (PIDs), are sorts of diseases caused by monogenic mutations, resulting in dysfunctions in immunity, and present vulnerable to infectious disease, autoimmune diseases, autoinflammatory diseases, and malignancies. Till now, more than 430 IEIs have been reported and classified into 10 categories [1]. For each category, they have unique clinical and immune characteristics, and are prone to different pathogen infections, indicating distinct underlying immunity defects [2, 3]. It’s a great challenge to diagnose and treat these rare diseases. Characteristic clinical manifestations, including typical infectious spectrum, will provide clues for disease recognition and diagnosis.

*Talaromyces marneffei* (TM, formerly known as *Penicillium marneffei*), is an important opportunistic pathogen and can result in local or disseminated talaromycosis in immunocompromised individuals. This is a dimorphic fungus, growing as a mold at 25 °C, and as a yeast at 37 °C [4]. The morphological conversion is associated with its virulence [5]. It was first isolated from bamboo rat in Vietnam in 1956 [6], and is mainly endemic in Southeast Asia, Northeast India, and South China [7–10]. Typical symptoms of disseminated talaromycosis include fever, anemia, weight loss, hepatosplenomegaly, lymphadenopathy, and multiply organ involvements [11, 12]. In spite of antifungal treatments, the mortality of talaromycosis remains high [13].

TM infections are widely reported in human immunodeficiency virus (HIV)-infected adults, especially in those with severely decreased CD4+ T cells (less than 100 cells/μL) [14], implying the role of cellular immunity against this fungus. Less commonly, TM infections are also described in people with secondary immunodeficiency conditions, such as malignancies [15], post-transplants [16], autoimmune diseases [17], and diabetes mellitus [18]. Talaromycosis is uncommon in otherwise healthy persons. Thus, TM infections in persons who are HIV negative and without secondary immunodeficiencies may suggest underlying primary immunodeficiencies, that is, IEIs. Actually, some HIV-negative adults with TM infections were detected with anti-IFN-*γ* autoantibodies, belonging to a kind of adult-onset IEIs [19, 20]. Most recently-reported children patients are also HIV-negative, and more infected children were revealed to have IEIs, such as hyperimmunoglobulin M syndrome (HIGM) [21], hyper-IgE syndrome (HIES) [22, 23], and STAT1 gain-of-function (GOF) disorder [24]. However, the reported spectrum of IEIs with TM infections is very limited. The clinical and immunological data on HIV-negative children with TM infections are even scarcer.

In this study, we retrospectively reviewed 8 children with TM infections and all of them were finally diagnosed as IEIs. Analysis of their clinical, immunological and genetic features may increase pediatricians’ awareness of this fungus infection. TM infections in HIV-negative children could help to diagnose different categories of IEIs. Besides, the spectrum of IEIs with TM infections may in reverse shed light on the underlying mechanisms for anti-TM infections.

## Materials and Methods

### Patients and Clinical Data

A total of 8 patients with TM infections were identified in Shenzhen Children’s Hospital during 2017–2021. All of the patients were HIV-negative. Clinical data were collected from patients’ medical records, including demographic information, initial clinical symptoms, onset age, family histories of IEIs, recurrent infections, complications, physical examinations, routine laboratory examinations, and follow-ups. Informed consents were obtained from each patients’ parents or guardians. This retrospective study was approved by the Ethics Committee of Shenzhen Children’s Hospital (2019050).

### TM Identification

#### Microbiological Culture

Specimens including blood, bone marrow (BM), sputum, bronchoalveolar lavage fluid (BALF), ascites, cerebrospinal fluid (CSF), lung biopsies, or lymph nodes biopsies, were cultured on Sabouraud dextrose agar at 25 °C. Typical TM colonies present flat green-yellowish surfaces and produce diffusible red pigments to the surroundings. Multinucleate hyphal can be observed under microscope and cells divide by septation. When incubated at 35 °C, the conidia convert to yeasts and divide by medial fission.

#### Histopathological Examination

Wrights-stained bone marrow smears were observed under microscope. In positive infections, numerous yeast-like cells were observed inside and outside host cells. The yeast-likes cells were 2–4 μm in diameter and had clear transverse septa. Tissue sections from lymph node biopsy were stained with hematoxylin and eosin (H&E), Grocott methenamine silver (GMS), and periodic acid–Schiff (PAS) stain. Positive sections showed that small yeast-like cells distributed within macrophages and histiocytes, and divided by fission.

#### Megagenomic Next-Generation Sequencing (mNGS)

DNA in BALF was extracted using TIANamp Micro DNA Kit (TIANGEN, Beijing, China). DNA libraries were constructed through DNA-fragmentation, end-repair, adapter-ligation and PCR amplification. Library qualities were analysed using Agilent 2100 and then qualified libraries were sequenced using MGISEQ-2000 platform by BGI (Shenzhen, China). Then low-quality reads were trimmed off. Human host sequences which were mapped to human reference genome (hg19) using Burrows-Wheeler Alignment (BWA) were computational subtracted [25]. The remaining data were classified by simultaneously aligning to four Microbial Genome Databases, consisting of bacteria, fungi, viruses and parasites. The classification reference databases were downloaded from NCBI (ftp://ftp.ncbi.nlm.nih.gov/genomes/).

### Genetic Analysis

Genomic DNA was extracted from peripheral blood mononuclear cells (PBMCs) of patients and their parents using QIAamp DNA Mini Kit (Qiagen, Germany). Whole exome sequencing or targeted panel sequencing were performed using Illumina HiSeq 2000 by MyGenostics (Beijing, China). Targeted panel was able to capture 423 IEI-related genes using the GenCap custom enrichment kit (MyGenostics). Then the qualified paired-end clean reads were mapped to the human reference genome (hg19). Duplicated reads were removed using Picard (http://broadinstitute.github.io/picard/). Variants were called by genome analysis toolkit (GATK) pipeline [26]. The identified variants were annotated using ANNOVAR [27], associated with 1000 genomes, Exome Aggregation Consortium, and the Human Gene Mutation Database. REVEL (rare exome variant ensemble learner) was used for functional prediction [28]. Splice site was predicted by Human Splicing Finder [29]. The pathogenicity of mutations was assessed following the American College of Medical Genetics and Genomics guideline [30]. All putative pathogenic variants were confirmed by Sanger sequencing.

### Flow Cytometry Analysis

#### Proportion of IL-17A-Producing Cells

PBMCs were stimulated by Cell Stimulation and Protein Transport Inhibitor Cocktail (2 μl/ml, Biogems, USA) for 4 h at 37 °C. Then, PBMCs were washed twice with phosphate buffer saline (PBS) and incubated with CD3-PerCP-Cy5.5 and CD8-BV510 (BD Biosciences, USA) for 20 min at room temperature. Cells were then washed twice with PBS, and fixed with Intracellular Fixation Buffer (eBioscience, USA) for 30 min at 4 °C. After fixation, cells were permeabilized with diluted Permeabilization Buffer (1 × , eBioscience, USA) for 20 min at room temperature. Cells were then washed twice and incubated with IL-17A-PE (eBioscience, USA) for 30 min at room temperature. Cells were then washed twice and resuspended and acquired on BD FACS Canto II flow cytometer.

#### Lymphocyte Subsets

Immunophenotyping of peripheral blood lymphocyte subsets were mainly based on Reference [31]. Briefly, EDTA-anticoagulated whole blood was split into 2 parts and stained with different panels of antibodies to explore T cells and B cells separately. For T cell subsets, the antibody panel contained CD3-PerCP-Cy5.5, CD4-FITC, CD8-BV510, CD45RA-PE-Cy7, CD27-APC, TCR aβ-PE, and TCR γδ-BV421. For B cell subsets, the antibody panel contained CD19-APC, CD27-V450, IgD-BV510, CD24-PE, and CD38-PerCP-Cy5.5 (All antibodies were purchased from BD Biosciences). After 20 min, erythrocytes in blood sample were lysed by red cell lysis buffer (TIANGEN, China). Then cells were centrifuged and washed twice with PBS. After resuspended, the cells were acquired on BD FACS Canto II, and the data were analyzed using FlowJo (V10.4, BD Biosciences).

#### CD40 Ligand (CD40L) Expression

PBMCs were stimulated by 50 ng/mL phorbol myristate acetate (PMA, Merck) and 500 ng/mL ionomycin (Merck) for 4 h at 37 °C. After washed twice with PBS, cells were stained with CD4-PerCP-Cy5.5 (BD Biosciences, USA) and CD40L-FITC (eBioscience, USA) for 30 min at room temperature. Then cells were washed twice with PBS and resuspended and acquired on BD FACS Canto II flow cytometer. The percentage of CD4 + CD40L + cells were determined.

### Search Strategy for Literature Review

PubMed was systematically searched for English articles published from 1956 to 2022. China National Knowledge Infrastructure (CNKI) (http://cnki.net/) and Wanfang (http://www.wanfangdata.com.cn/) data were also searched for Chinese articles published during the same period. Search terms were (“Talaromyces marneffei” OR “Penicillium marneffei” OR “marneffei” OR “penicilliosis” OR “talaromycosis”) AND (“mutation” OR “immunodeficiency” OR “inborn errors of immunity”). Their Chinese equivalents were used for Chinese database search. Full-text version of the original articles were reviewed. Exclusion criteria were as follows: (1) The patients were HIV-positive; (2) No detailed mutation information was provided; (3) The reported mutations were unrelated to IEIs; (4) There was no convinced evidence for TM infections.

### Statistical Analysis

Continuous data which were not in Gaussian distributions were described as median (range). Categorical data were presented as numbers and percentages.

## Results

### Identification of TM Infections

During 2017–2021, eight children with TM infections were identified in Shenzhen Children’s Hospital, and all of them had confirmed microbiological evidences. Seven patients had positive culture results, using specimens from blood (P2, P3, P5), sputum (P2, P4, P5), BALF (P2, P4), BM (P4, P5), CSF (P4), ascites (P6), lung biopsy (P1), and lymph node biopsy (P8), separately. Typical images of TM infection were observed in BM smears from P2 and P5. mNGS for BALF reinforced TM infections in P8. For P7, histopathological examination of the lymph node tissue revealed granulomatous inflammation. PAS and GMS staining showed that numerous yeast-like or sausage-like organisms distributed within macrophages, and had transverse septa, demonstrating TM infection.

### Clinical Manifestations

This study included 2 females and 6 males, with a median onset age of TM infections at 23 months old (range: 4–102 m). All the 8 patients were born and lived in the southeast of China, including 5 patients in Guangdong, 1 patient in Hunan, 1 patient in Yunnan, and 1 patient in Hubei province (Table 1).

**Table 1:** Clinical features of the 8 patients with TM infection

Patients	Gender	TM Onset age	Medical history	Symptoms/signs	Concurrent infections	Genetic defect	Outcome
P1	F	7y11m	1y3m: thrush, diarrhea, severe pneumonia, disseminated tuberculosis	Weight loss, fever, skin lesions, CMC, pneumonia, hepatosplenomegaly, lymphadenopathy, anemia	*M.* *catarrhalis, H* *.influenzae,* *S.* *pneumoniae,* EBV, rotavirus	*STAT1* (WES)	Improved
P2	M	1y5m	1y3m: lymphatic tuberculosis	Weight loss, fever, pneumonia, ARDS, thrush, diarrhea, hepatomegaly, hepatic failure, pancytopenia	CMV, *K.* *pneumoniae,* *E.* *cloacae,* *B.* *cepacia*	*ADA* (IEI panel)	Death
P3	M	1y1m	5 m:axillary abscess; 6 m: severe pneumonia, ARDS, eosinophilia, CMV infections	Weight loss, fever, pneumonia, abdominal distension, diarrhea, hematochezia, jaundice, hypothyroidism, hapatosplenomegaly, lymphadenopathy, portal hypertension, cavernous transformation of the portal vein anemia	CMV, *S.* *typhimurium*	*CD40LG* (IEI panel)	Death
P4	F	2y5m	–	Weight loss, fever, disturbance of consciousness, intracranial infection, cerebral infarction, cerebral hernia, pneumonia, respiratory failure, lymphadenopathy, skin lesions, anemia	*M.* *pneumoniae*	*STAT3* (IEI panel)	Death
P5	M	8 m	3 m: PJP Family history: One brother died from TM infection at 6 m	Fever, pneumonia, rash, edema, hematuresis, diarrhea, hepatosplenomegaly, anemia, thrombocytopenia	Rhinovirus	*IL2RG* (IEI panel)	Death
P6	M	4 m	alpha thalassaemia	Weight loss, fever, pneumonia, peritonitis, hepatosplenomegaly, pancytopenia, HLH, MODS,	Disseminated tuberculosis, *C.* *parapsilosis,* rhinovirus	*IL2RG* (WES)	Death
P7	M	4y7m	–	Weight loss, fever, lymphadenopathy, hepatosplenomegaly	–	*STAT1* (WES)	Improved
P8	M	8y6m	–	Weight loss, fever, pneumonia, osteolytic lesions, lymphadenopathy, hepatosplenomegaly, lymphopenia, CMC	*C.* *albicans*, Rhinovirus, S. *aureus,* *M.* *catarrhalis, H.* *influenzae*	*STAT1* (WES)	Improved

*TM T. marneffei*, *F* female, *M* male, *CMV* cytomegalovirus, *EBV* Epstein-Barr virus, *PJP* pneumocystis jiroveci pneumonia, *CMC* chronic mucocutaneous candidiasis, *ARDS* acute respiratory distress syndrome, *HLH* hemophagocytic lymphohistiocytosis, *MODS* multiple organ dysfunction syndrome, *WES* whole exome sequencing, *IEI* inborn errors of immunity

All the 8 patients presented disseminated talaromycosis. The most common symptom of TM infections was fever (8/8), followed by weight loss (7/8), pneumonia (7/8), hepatomegaly (7/8), splenomegaly (6/8), anemia (6/8), lymphadenopathy (5/8), thrombocytopenia (3/8), diarrhea (3/8), rashes or skin lesions (3/8), and osteolytic lesions (1/8) (Table 1). Besides, eosinophilia was observed in P2 and P3 (Table S1).

There were life-threatening complications, including acute respiratory distress syndrome (ARDS) and hepatic failure (P2), cerebral infarction and hernia, and respiratory failure (P4), portal hypertension (P3), hemophagocytic lymphohistiocytosis (HLH) and multiple organ dysfunction syndrome (MODS) (P6). Six patients experienced episodes in intensive care unit.

Special pathogens need to be noticed. Three patients had previous or concurrent *M.tuberculosis* (TB) infections (P1, P2, and P6), two of whom with disseminated tuberculosis. P5 had *Pneumocystis jiroveci* pneumonia (PJP) at 3 months old. P1 and P8 had chronic mucocutaneous candidiasis, which caused by *C.* *albicans.* P3 had recurrent *S.* *typhimurium* enteritis, proven by positive stool culture. Three patients were co-infected by herpes virus. Epstein-Barr virus (EBV) and cytomegalovirus (CMV) DNAs were positively amplified in BALFs from P1 and P2, separately. CMV DNAs were detected in serum and urine in P3 and CMV-specific IgM antibodies were also positive. Bacteria including *M.catarrhalis* and *H.influenzae* were also found concurrent with TM infections.

For laboratory examinations (Table S1), most patients had elevated erythrocyte sedimentation rate (ESR) (5/6) and c-reactive protein (CRP) (7/8). 3/6 patients had increased procalcitonin. Serum 1,3-beta-D-glucan was elevated in 5/7 patients. Higher aspartate amino transferase (AST) was observed in 5/8 patients. Serum lactate dehydrogenase (LDH) was elevated in 5/8 patients. As for conventional immunological assessments, CD3+ T cells were decreased in 6 patients (except for P3 and P7), together with decreased CD4+ and CD8+ T cells. Reduced CD19+ B cells were only observed in P2 and P8. All the patients had decreased natural killer (NK) cells.

Autoimmunity was found in four patients. P1 presented positive antinuclear antibodies, perinuclear antineutrophil cytoplasmic antibodies (pANCA), and autoantibodies to thyroglobulin, thyroid peroxidase, SS-A, ribonucleoprotein, and platelet. Another three patients (P3, P4, P8) exhibited autoantibodies and positive Coombs test. Low complement C3 level was observed in P2.

### Genetic Analysis and IEI Diagnosis

**Fig. 1:**
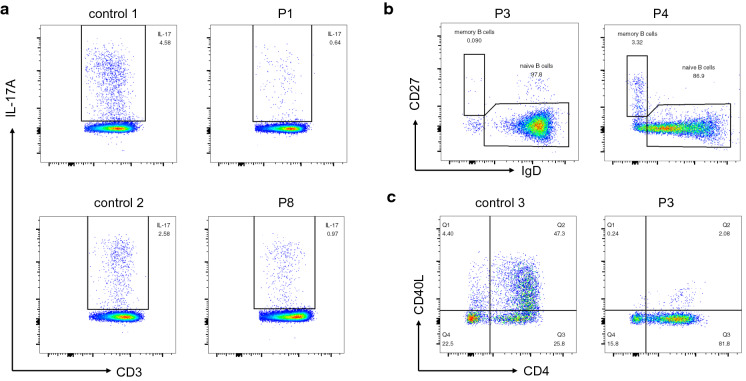
Flow cytometric analysis of immunological functions in patients with T.marneffei infections. **a** Proportions of CD3 + CD8-IL-17A + T cells following stimulation with PMA and ionomycin were reduced in P1 and P8. **b** Percentages of CD19 + IgD-CD27 + memory B cells were decreased in P3 and P4. **c** The expression of CD40 ligand in CD4+ T cells stimulated by PMA and ionomycin was dramatically reduced in P3

Based on the clinical symptoms and conventional immunological tests, all 8 patients further went gene sequencing (Table 1). Whole exome sequencing revealed heterozygous mutations in *STAT1* in three patients: L351F in P1, D65N in P7, and M390I in P8. All these three missense mutations have been reported to cause STAT1 GOF mutations [24, 32, 33]. We further examined the proportion of IL-17A-producing T cells in P1 and P8, and found out that patients had decreased IL-17A-producing T cells (Fig. 1a).

The other five mutated genes found in patients were related to combined immunodeficiency (CID). Nonsense mutation W155X in *IL2RG* was found in P5 and P6, leading to X-linked severe combined immune deficiency (SCID). These two boys had lymphopenia, with very low CD3+ T cells and NK cells, and high proportion of B cells. The immunoglobulin levels were very low. Compound heterozygous mutations in *ADA* were detected in P2. He had profound lymphopenia, very low CD3+ T cells, CD19+ B cells and NK cells, consistent with T-B-NK- SCID phenotype. The immunoglobulin levels were not detected before intravenous immunoglobulin (IVIG). P3 had large fragment deletion in *CD40LG*. The IgG and IgA concentrations were low while the IgM level was within the normal range. Flow cytometry revealed that CD19 + IgD-CD27 + memory B cells were nearly absent (0.09%, normal range: 2.98–14.18%, Fig. 1b). After stimulated by PMA and ionomycin, CD40L expression was lower in P3 than that in control (2.08% vs. 47.3%, Fig. 1c). P4 had a heterozygous missense mutation in *STAT3*. The proportion of CD19 + IgD-CD27 + memory B cells was reduced (3.32%, normal range: 3.6–18.55%, Fig. 1b).

### Treatments and Outcomes

Treatments were summarized in Table 2. All patients received anti-fungal drugs after TM suspicion or identification. Four of them were treated with additional anti-TB medications. One patient was diagnosed as HLH and treated with methylprednisolone and etoposide. Sulfamethoxazole and trimethoprim were added in 4 patients for PJP prophylaxis and IVIG were administered monthly in six patients. Besides medications, five patients ever had respiratory failure and were supported by mechanical ventilation. Four patients received fiberoptic bronchoscopy examination and bronchoalveolar lavage. Five patients had surgeries, including abscess excisions in P2 and P3, decompressive craniectomy in P4, and lymph nodes biopsies in P7 and P8.

**Table 2:** Treatments of IEI patients with TM infections

Patients	Antifungal	Anti-TB	Other treatments
P1	VCZ, AmB, ITZ, FCZ	INH, RFP, PZA, LNZ	IVIG
P2	VCZ, AmB	INH, RFP	CoSMZ, GCV, IVIG
P3	VCZ, ITZ, AmB	–	CoSMZ, GCV, IVIG
P4	VCZ, MCFG, AmB, ITZ	INH, RFP, PZA	ACV, DXM, IVIG
P5	VCZ, AmB, ITZ	–	CoSMZ, IVIG
P6	VCZ	INH, RFP, PZA, LNZ	MP, VP-16, CoSMZ, IVIG
P7	AmB, ITZ	–	–
P8	VCZ, ITZ	–	–

*IEI* inborn errors of immunity, *TM T.* *marneffei*, *AmB* amphotericin B, *VCZ* voriconazole, *ITZ* itraconazole, *FCZ* fluconazole, *MCFG* micafungin, *INH* isoniazid, *RFP* rifampicin, *PZA* pyrazinamide, *LNZ* linezolid, *CoSMZ* compound sulfamethoxazole and trimethoprim, *AZM* azithromycin, *GCV* ganciclovir, *ACV* acyclovir, *DXM* dexamethasone, *MP* methylprednisolone, *VP-16* etoposide, *IVIG* intravenous immunoglobulin

As for outcomes, 3 patients with *STAT1* GOF mutations had improved. They were followed-up regularly and received itraconazole or fluconazole for long-term prophylaxis. The other 5 patients presenting combined immunodeficiencies, including SCID, HIGM, HIES, died although after comprehensive therapy.

## Discussion

TM is an endemic opportunistic fungus, leading to multi-organ damages and poor prognosis in immunocompromised individuals. TM infections in children were rare. Most children patients were HIV-negative and more children patients were diagnosed as IEIs. In this study, we identified 8 children with TM infections, and finally all the patients were diagnosed as IEIs. We described TM infections in an adenosine deaminase (ADA) deficient child for the first time and enriched the spectrum of IEIs underlying TM infections.

Till now, 9 types of IEIs have been reported with TM infections, that is, CD40 ligand deficiency (12 cases), STAT1 GOF (11 cases), STAT3 deficiency (7 cases), X-SCID (3 cases), IFN-*γ* receptor 1 deficiency (2 cases), CARD9 deficiency (2 cases), ADA deficiency (1 case), RelB deficiency (1 case) and NFKB2 deficiency (1 case) (Table 3). There were more IEI patients reported with TM infections, however, the detailed mutations were not described. Thus, these cases were not included. For *STAT1* GOF mutations, all the published cases were missense mutations. For *CD40LG* gene, deletions, especially large fragment deletions, and splice site mutations, were more frequently reported. Missense and in-frame deletions were common mutations in STAT3 deficiencies. The currently reported two cases with *CARD9* mutations were both compound heterozygous mutations. All the three X-SCID patients had nonsense mutations in *IL2RG*, and died during the follow-ups.

**Table 3:** Reported inborn errors of immunity in HIV-negative children with *T. marneffei* infection

Patient number	Geneticdefect	Inheritance	Nucleotide change	Amino acid change	Mutation type	Outcome	References
P1	*STAT1*	AD GOF	c.1053G > T	p.L351F	Missense	Improved	This study
P2	*ADA*	AR	(1) c.730delG (2) c.202T > A	(1) p.E244KfsX67 (2) p.Y68N	Compound heterozygous mutations	Dead	This study
P3	*CD40LG*	XL	–	–	Large fragment deletion including exon1-5	Dead	This study
P4	*STAT3*	AD LOF	c.115G > A	p.E39K	Missense	Dead	This study
P5	*IL2RG*	XL	c.464G > A	p.W155X	Nonsense	Dead	This study
P6	*IL2RG*	XL	c.464G > A	p.W155X	Nonsense	Dead	This study
P7	*STAT1*	AD GOF	c.193G > A	p.D65N	Missense	Improved	This study
P8	*STAT1*	AD GOF	c.1170G > A	p.M390I	Missense	Improved	This study
P9	*STAT1*	AD GOF	c.800C > T	p.A267V	Missense	Improved	[32]
P10	*STAT1*	AD GOF	c.821G > A	p.R274Q	Missense	Improved	[42]
P11 (adult)	*STAT1*	AD GOF	c.859 T > A	p.Y287N	Missense	Improved	[60]
P12	*STAT1*	AD GOF	c.863C > T	p.T288I	Missense	Improved	[32]
P13	*STAT1*	AD GOF	c.1053G > T	p.L351F	Missense	Improved	[24]
P14	*STAT1*	AD GOF	c.1074G > T	p.L358F	Missense	Dead	[32]
P15	*STAT1*	AD GOF	c.1170G > A	p.M390I	Missense	Improved	[32]
P16	*STAT1*	AD GOF	c.193G > A	p.D65N	Missense	Improved	[24]
P17	*STAT3*	AD LOF	c.1679_1681del	Not stated	Deletion	Improved	[21]
P18	*STAT3*	AD LOF	c.1593A > T	Not stated	Missense	Improved	[21]
P19	*STAT3*	AD LOF	c.1593A > T	p.K531N	Missense	Improved	[23]
P20 (adult)	*STAT3*	AD LOF	c.92G > A	p.R31Q	Missense	Improved	[61]
P21	*STAT3*	AD LOF	c.1121A > G	p.D374G	Missense	Improved	[62]
P22	*STAT3*	AD LOF	c.1673G > A	p. G558D	Missense	Improved	[22]
P23	*CD40LG*	XL	c.424_436del	Not stated	Deletion	Improved	[21]
P24	*CD40LG*	XL	> 132 kb	Not stated	Large fragment deletion	Improved	[21]
P25	*CD40LG*	XL	c.1978 + 1G > A	Not stated	Splicing error	Improved	[21]
P26	*CD40LG*	XL	c.598A > T	Not stated	Missense	Improved	[21]
P27	*CD40LG*	XL	g.IVS1-3 T > G (c.157insAG)	p.I53RfsX2	Splicing error	Dead	[63]
P28	*CD40LG*	XL	g.IVS1 + 1G > A (c.75-156del82bp)	p.M25IfsX26	Splicing error	Improved	[63]
P29	*CD40LG*	XL	g.IVS3 + 1G > A (exon3 missing)	–	Splicing error	Improved	[63]
P30	*CD40LG*	XL	g.IVS1-1G > A (c.158-161delTAGA)	p.I53KfsX13	Splicing error	Lost to follow-up	[63]
P31	*CD40LG*	XL	g.IVS4 + 1G > C (exon4 missing)	p.L116-136del	Splicing error	Improved	[63]
P32	*CD40LG*	XL	–	–	Large fragment deletion including exon4-5	Improved	[63]
P33	*CD40LG*	XL	g.IVS1 + 1G > A	p.M25IfsX26	Splicing error	Improved	[32]
P34	*IL2RG*	XL	c.185G > A	Not stated	Nonsense	Dead	[21]
P35	*CARD9*	AR	(1) c.440 T > C (2) c.586A > G	(1)p.L147P (2)p.K196E	Compound heterozygous mutations	Dead	[40]
P36	*CARD9*	AR	(1) c.1118G > C (2) c.610C > T	(1)p.R373P (2)p.R204C	Compound heterozygous mutations	Improved	[39]
P37	*IFNGR1*	AR	c.182dupT	p.V61fsX69	Insertion	Dead	[32]
P38	*IFNGR1*	AR	c.182dupT	p.V61fsX69	Insertion	Improved	[32]
P39	*RELB*	AR	c.400_401insAGC	p.Q135_R136insQ	Insertion	Improved	[41]
P40	*NFKB2*	AD	c.2540dupT	–	Insertion	Improved	[42]

*HIV* human immunodeficiency virus, *AD* autosomal dominant, *AR* autosomal recessive, *XL* X-linked, *GOF* gain-of-function

Although the invasion mechanisms were not clearly deciphered, it was widely believed that TM infections initiated from the inhalation of conidia [34, 35]. Then the conidia converted into yeast phase and were engulfed by macrophages [36]. The pathogenic yeasts could survive and replicate within macrophages, and even disseminated when the hosts were in immunocompromised situation [37]. Therefore, efficient macrophage activation and functions were key factors for anti-TM strategies. CD4+ T cells activated macrophages by offering CD40L-CD40 interactions and secreting IFN-*γ* [38]. Thus, CD4+ T lymphopenia, CD40 ligand absence, or interfered IFN-*γ* pathway could hamper macrophages to eliminate intracellular TM. HIV infections, *IL2RG* and *ADA* mutations all led to remarkable reduced CD4 + T cells. CD40 ligand deficiency impaired T cell-antigen presenting cell (APC) interactions. Autoantibodies against IFN-*γ* and IFN-*γ* receptor 1 deficiency blocked IFN-*γ* signaling. These defects were all reported to associate with TM infections.

On the other hand, patients with STAT1 GOF and STAT3 deficiency presented impaired Th17 responses [1], indicating that IL-17 signaling might be involved in anti-TM immunity. In addition, NF-κB (nuclear factor kappa light chain enhancer of activated B cells) pathways may also contribute to host strategies against TM infections. Patients with *CARD9* [39, 40], *RELB* [41]*,* or *NFKB2* [42] mutations have been reported with TM infections. As an adaptor protein, CARD9 mediated signals from pattern recognition receptors (PRRs) which could recognize TM-related carbohydrates, to the downstream transcription factor NF-κB [43]. CARD9 deficiency impaired NF-κB activation, cytokines secretion, Th17 differentiation, and neutrophil killing [44, 45]. RelB and NF-κB2 are components of NF-κB family. RelB deficiency led to reduced T cell proliferations to mitogens and skewed T cell receptor (TCR) repertoire, together with impaired antibody responses, presenting combined immunodeficiency [46]. NFKB2 deficiency resulted in damaged B cell differentiation and hypogammaglobinemia. A series of lymphocyte subpopulations, including regulatory T cells, Th17 cells, and circulating T follicular helper cells were also decreased [47]. More cases and experiments are needed to demonstrate the roles of NF-κB pathway in TM infections.

We noticed reduced NK cells in all the eight patients in our study. The reported TM-infected children with *CARD9*, *IFNGR1*, or *RELB* mutations, also exhibited decreased NK cells [32, 39–41]. Zeng et al. [21] reported reduced NK cells in 62% of the TM-infected patients. In Guo’s study, 81.8% of the children with TM-infections exhibited decreased NK cells [48]. NK cells from patients with STAT1 GOF mutations displayed decreased IFN-*γ* production and reduced proliferation after stimulation of IL-15 [49]. NK cell was another important source of IFN-*γ* secretion, and participated in host responses against various pathogens, especially for herpes virus and intracellular bacteria [50, 51]. In our patients, EBV and CMV were observed in three TM-infected patients.

TM infection was a severe disease with high mortality. In our study, 5 patients died during the follow-ups (62.5%). Zeng et al. [21] reported 11 in 21 children (52.38%) died of TM infections. In Guo’s study, they enrolled 11 TM-infected HIV-negative children and four of them (36.36%) died during 1-year follow-ups [48]. Early recognition and comprehensive therapy may help to improve the prognosis.

Currently, guidelines recommended initial treatments for HIV-associated TM infections in adults with amphotericin B deoxycholate at a dose of 0.7–1 mg/kg/day for 2 weeks, followed by itraconazole at a dose of 400 mg per day for 10 weeks [52]. Considering the substantial side effects and limited availability of amphotericin B, other anti-fungal drugs were also used. The trial conducted by Le et al. proved that amphotericin B was superior to itraconazole as induction therapy for HIV-associated talaromycosis [53]. Huang’s trial indicated that voriconazole was noninferior to amphotericin B as an induction antifungal drug for HIV- associated disseminated talaromycosis [54]. There were also evidences supporting the use of amphotericin B, itraconazole, voriconazole, and posaconazole in management of TM infections, not fluconazole [55]. For TM infections in HIV-negative children patients, the treatments were still lacking consensus and remained a big challenge. Most of the infected children had underlying IEIs. They were susceptible to various pathogens except for TM and anti-bacteria and anti-virus therapy were necessary. Especially, intracellular bacteria, including mycobacteria and salmonella, were commonly co-infected with TM [19, 56], as hosts shared some core strategies to defend these intracellular pathogens. People also discussed the prophylaxis for TM infections [57–59]. For IEIs listed in Table 3, prophylactic anti-fungal drugs may be given to gain clinical benefits.

In conclusion, we retrospectively analysed TM infections in 8 Chinese children and all of them were diagnosed as IEIs, including STAT1 GOF, IL-2 receptor common gamma chain deficiency, ADA deficiency, CD40 ligand deficiency, and STAT3 deficiency. Based on our patients and literature review, the spectrum of IEIs underlying TM infections indicated that T cell-mediated immunity, IFN-*γ*, IL-17 signalings and NF-κB pathways were important for host responses against TM infection. In reverse, for children born or living in South China or other endemic areas, without HIV infection, without other secondary immunodeficiency, TM infections may be an indicator for IEIs and further immunological and genetical evaluations are needed.

## Supplementary Information

Below is the link to the electronic supplementary material.

Supplementary file1 (DOCX 27 KB)
