# Supplementary material for: Talaromyces marneffei Infections in 8 Chinese Children with Inborn Errors of Immunity
Source: Mycopathologia. 2022 Sep 30;187(5-6):455–67. doi: 10.1007/s11046-022-00659-0 (PMC9524311; doi:10.1007/s11046-022-00659-0)
Supplement: Supplementary file 1 — Supplementary file1 (DOCX 27 KB) [file 11046_2022_659_MOESM1_ESM.docx]

Table s1. Laboratory findings of the children with *T. marneffei* infections

|  | P1 | P2 | P3 | P4 | P5 | P6 | P7 | P8 |
| --- | --- | --- | --- | --- | --- | --- | --- | --- |
| WCC(/uL) | 7020 | 2760 | 9980 | 12050 | 5190 | 4450 | 12000 | 6800 |
| ANC(/uL) | 5260 | 1880 | 2800 | 8240 | 3660 | 3640 | 6900 | 5800 |
| ALC(/uL) | 1410 | 470 | 3810 | 3040 | 1280 | 580 | 3860 | 530 |
| PLT(*1000/uL) | 349 | 12 | 150 | 385 | 9 | 20 | 363 | 371 |
| HB（g/L） | 102 | 81 | 98 | 100 | 86 | 69 | 118 | 112 |
| ESR(mm/h) | 30 | NA | 57 | 104 | NA | 8 | 41 | 50 |
| CRP(mg/L) | 35.5 | 38.7 | 43.5 | 24.6 | 3.1 | 124.3 | 21.19 | 30.96 |
| PCT(ng/mL) | NA | 0.34 | 27.44 | 0.06 | 0.71 | 1.38 | 0.11 | NA |
| 1,3-beta-D-glucan assay(pg/mL) | 90.65 | 492.28 | 420.4 | 43.76 | <37.5 | NA | 100.1 | 115.93 |
| LDH(IU/mL) | 379 | 1099 | 303 | 508 | 261 | 418 | 294 | 272 |
| AST(IU/mL) | 47 | 194 | 140 | 19 | 181 | 202 | 44 | 29 |
| IgG(g/L) | 34.76 | 21.15^a^ | 0.37↓ | 29.03 | 2.32↓ | 2.72↓ | 19.91 | 24.36 |
| IgA(g/L) | 2.83 | 0.56 ^a^ | 0.04↓ | 1.29 | 0.01↓ | 0.06↓ | 3.53 | 3.7 |
| IgM(g/L) | 1.22 | 2.06 ^a^ | 1.21 | 2.15 | 0.02↓ | 0.05↓ | 1.08 | 1.09 |
| CD3+(/ul)(%) | 741(62.78)↓ | 410(87.2) ↓ | 2440 (80.87) | 1035 (33.89)↓ | 247 (6.03) ↓ | 199 (35.34) ↓ | 1902.4 (82) | 433 (76.74) ↓ |
| CD3+ normal range for age and gender^b^ | 1480-2847 (59.5-75.56) | 1794-4247 (53.88-72.87) | 1794-4247 (53.88-72.87) | 1775-3953 (53.37-71.91) | 2187-6352 (55.32-73.11) | 2179-4424 (54.28-71.67) | 1424-2664 (60.05-74.08) | 1325-2276 (57.10-73.43) |
| CD4+(/ul)(%) | 501 (41.16) ↓ | 146.6 (31.2) ↓ | 1751 (54.88) | 711 (23.12) ↓ | 67 (1.63) ↓ | 6 (1.07) ↓ | 997.6 (43) | 187 (33.77) ↓ |
| CD4+ normal range for age and gender^b^ | 767-1592 (28.49-41.07) | 902-2253 (24.08-42.52) | 902-2253 (24.08-42.52) | 948-2477 (26.19-45.48) | 1125-3768 (28.17-47.74) | 1461-3018 (33.72-52.43) | 686-1358 (26.17-40.76) | 531-1110 (24-38.72) |
| CD8+(/ul)(%) | 178 (14.63) ↓ | 143.3 (30.5) ↓ | 740 (23.18) | 282 (9.18) ↓ | 184 (4.46) ↓ | 186 (33.71) ↓ | 765.6 (33) | 141 (25.48) ↓ |
| CD8+ normal range for age and gender^b^ | 553-1127 (19.7-32.04) | 580-1735 (19.0-32.51) | 580-1735 (19.0-32.51) | 531-1521 (16.29-29.88) | 686-2278 (15.88-31.48) | 556-1687 (14.08-24.7) | 518-1125 (19.68-34.06) | 480-1112 (21.01-33.94) |
| CD19+(/ul)(%) | 332 (29.00) | 10.67 (2.27) ↓ | 438 (15.4) | 1952 (64.34) | 3708 (91.46) | 328 (57.43) | 301.6 (13) | 89 (14.06) ↓ |
| CD19+ normal range for age and gender^b^ | 303.52-777.25 (10.46-21.77) | 461-1456 (13.23-26.39) | 461-1456 (13.23-26.39) | 537.11-1464.39 (13.93-30.49) | 916-1832 (17.2-29.71) | 734-2265 (17.34-36.03) | 280-623 (10.21-20.12) | 216-536 (9.19-19.48) |
| CD16/56+(/ul)(%) | 61 (5.3) ↓ | 44.37 (9.44) ↓ | 56 (1.96) ↓ | 113 (3.74) ↓ | 76 (1.87) ↓ | 28 (4.98) ↓ | 116 (5) ↓ | 20 (3.45) ↓ |
| CD16/56+ normal range for age and gender^b^ | 227-668 (7.83-20.99) | 270-1053 (7.21-20.9) | 270-1053 (7.21-20.9) | 241-978 (6.53-22.24) | 306-896 (5.67-15.9) | 290-780 (5.89-14.85) | 258-727  (9-22.24) | 246-792 (10.01-26.98) |
| DHR flow cytometry test | Negtive | Negtive | Negtive | NA | NA | NA | NA | NA |
| ANA | 1：320, homogeneous | Negtive | NA | Negtive | NA | NA | Negtive | Negtive |
| Others | Auto-Abs to Tg, TPO, SS-A, pANCA, RNP, and platelet | EO:9.4%;  Decreased C3 | EO:25.5%;  Coombs++, Auto-Abs to Tg, TPO | Coombs++;  Auto-Abs to Tg, TPO |  |  |  | Auto-Abs to Tg, TPO |

^a^ Concentrations after intravenous immunogloblin

^b^ Normal ranges for age and gender are based on Reference [31]

Abbreviations: WCC: white cell count, ANC: absolute neutrophil count, ALC: absolute lymphocyte count, PLT: platelet, HB: hemoglobin, ESR: erythrocyte sedimentation rate, CRP: c-reactive protein，PCT: procalcitonin, LDH: lactate dehydrogenase, AST: aspartate amino transferase, DHR: dihydrorhodamine, ANA: antinuclear antibodies, EO: eosinophil, auto-Abs: auto-antibodies, Tg: thyroglobulin, TPO: thyroid peroxidase, NA: not available.
